# Current News

**Published:** 1895-05

**Authors:** 


### Current News.

### ANNUAL MEETING OF THE HARVARD ODONTOLOGICAL SOCIETY.

The Seventeenth Annual Meeting of the Harvard Odontological Society was held at Young's Hotel, Boston, February 23, at five o'clock, the President, Forrest G. Eddy, D.M.D., of Providence in the chair.

Reports were made by the recording and corresponding secretaries, treasurer, and chairman of the Committee on Clinics. This committee was appointed last March as an experiment, but since that time it has thoroughly demonstrated its usefulness, and has now become an important department of the Society. Six clinics have been given, five by members of the Society, as follows:

"Restoring to full contour an approximal surface of a bicuspid, using a steel matrix."

"Showing several cases of practical bridge-work."

"How to bake porcelain inlays."

"How to grind porcelain contours."

"The use of cocaine in the extirpation of the dental pulp."

"Method of burnishing gold in filling and restoring contours in an upper central."

The recording secretary reported the number of active members as forty-four and corresponding as thirteen.

The Society has held ten meetings during the past year, nine regular and one annual. Eleven essays have been presented, of which eight were contributed by members of the Society.

The December meeting was entirely devoted to the celebration of the "Semi-Centennial of the Discovery of Anæsthetics," and all members of the alumni were invited to be present, many accepted the invitation, and the occasion was most enjoyable.

An innovation was introduced this year by making the annual meeting "ladies night," and the response the invitation called out was very gratifying. After a social half-hour an adjournment was made to the banquet-hall, where forty-nine members and friends assembled. At the close of the dinner the orator of the evening, William P. Cooke, D.M.D., of Boston, held closely the attention of his hearers, taking as his theme "The Dentist as a Man."

The standard set by the orator was a high but not impracticable one. The dentist should make himself broad by a liberal education, seeking to train his whole mind just as the athlete does his whole body. The possibilities of the profession are great and the value of the work is in proportion to the service rendered.

A letter of regret from Professor Francis G. Peabody, D.D., of Cambridge was read, and also from Rev. W. E. Huntington, Ph.D., Dean, Boston University.

Interesting addresses were made by Mrs. Mary A. Livermore, Rev. W. B. Eddy, and John T. Codman, D.M.D.

The following officers were elected for the ensuing year: President, James Shepherd, D.M.D.; Recording Secretary, Waldo E. Boardman, D.M.D.; Corresponding Secretary, E. B. Hitchcock, D.M.D.; Treasurer, Dwight M. Clapp, D.M.D.; Editor, Henry L. Upham, D.M.D.; Orator for 1896, Edward S. Niles, D.M.D.

*Executive Committee.*—Waldo E. Boardman, D.M.D. (chairman), Arthur H. Stoddard, D.M.D., J. G. W. Werner, D.M.D.

James Shepherd,
*Corresponding Secretary.*

### DENTAL SOCIETY OF THE STATE OF NEW YORK.

The Twenty-seventh Annual Meeting of the above Society will be held in Academy Hall, 42 North Pearl Street, Albany, May 8 and 9, 1895. The subject for the meeting will be "Green-Stain," and divided as follows:

"The Etiology," illustrated with lantern slides, etc., by Carl Theodore Gramm, M.D., Chicago, Ill.

"The Classification," by W. C. Barrett, M.D., D.D.S., Buffalo.

"The Therapeutics," by S. B. Palmer, M.D.S., Syracuse.

Members of the profession are cordially invited to attend.

F. T. Van Woert, Brooklyn,
*President.*
C. S. Butler, Buffalo,
*Secretary.*

---

### NATIONAL ASSOCIATION OF DENTAL EXAMINERS.

It is earnestly requested of the presiding officers or secretaries of the Examining Boards throughout the States and Territories that they kindly forward to the National Secretary the full list of officers with their respective addresses.

In view of the large meeting expected at Asbury Park in August, the secretary desires to give every Board due notice in ample time, and likewise obtain a corrected list of officers to date.

Charles A. Meeker, D.D.S.,
*Secretary.*

29 Fulton Street, Newark, N. J.

---

### ILLINOIS STATE DENTAL SOCIETY.

The Thirty-first Annual Meeting of the Illinois State Dental Society will be held at Galesburg, May 14 to 17, inclusive. An interesting programme is in course of preparation. All dentists practising in Illinois are especially invited to attend. A cordial invitation is extended to the profession generally. This will be the first meeting in Galesburg since 1876, and it is the hope of the officers that it will be one of the most profitable meetings in the history of the Society.

Louis Ottofy,
*Secretary.*

Masonic Temple, Chicago.

